# A rare cause of gastrointestinal phytobezoars: diospyros lotus

**DOI:** 10.1186/1749-7922-7-19

**Published:** 2012-06-21

**Authors:** Gökhan Ertuğrul, Murat Coşkun, Mahsuni Sevinç, Behzat Yelimlieş, Fisun Ertuğrul, Toygar Toydemir

**Affiliations:** 1 Department of General Surgery, Düzce Atatürk State Hospital, Muncurlu, Düzce, Turkey; 2 Department of Anesthesiology and Reanimation, Düzce Atatürk State Hospital, Muncurlu, Düzce, Turkey; 3 Department of General Surgery, İstanbul Surgery Hospital, Nişantaşı, İstanbul, Turkey

**Keywords:** Gastrointestinal phytobezoars, Diospyros lotus (Persimmon)

## Abstract

**Aim:**

Diospyros Lotus (“Wild Date Palm of Trabzon or Persimmon”), which has been proven to cause phytobezoars, is a widely consumed fruit in the Black Sea and Northeast Anatolia regions of Turkey. The aim of the present study was to investigate the effects of Diospyros Lotus together with other predisposing factors, on the development of gastrointestinal phytobezoars and to discuss the treatment results in comparison to the literature.

**Material and method:**

The records of 13 patients, who had been admitted to the General Surgery Clinic of Düzce Atatürk State Hospital between August 2008 and August 2011, were retrospectively reviewed. Demographic characteristics, predisposing factors, clinical and radiological findings, diagnostic and therapeutic methods, and the outcomes of the patients were recorded from the patient files. Written informed consent was obtained from each patient for publication of this research article and accompanying images.

**Results:**

All the patients had a history of consuming Diospyros Lotus. Of the patients, 30,7% had a history of previous gastric surgery, 30,7% had diabetes mellitus and 23% had dental implants. None of the patients had hypothyroidism, which is another predisposing factor for phytobezoars.

The phytobezoars were located in the stomach alone in 23% of the patients, whereas 15,3% was detected in the jejunum and stomach, 15,3% was detected in the jejunum alone, and 46,1% was detected in the ileum alone. All patients were treated with surgery, and there were no deaths.

**Conclusion:**

Gastric phytobezoars are rare. Preventive measures have particular importance in the management of this condition, which is difficult to treat. For this purpose, excessive consumption of herbal nutrients containing a high amount of indigestible fibers such as Diospyros Lotus should be avoided in patients with a history of gastrointestinal surgery or poor oral and dental health.

## Introduction

**Figure 1:**
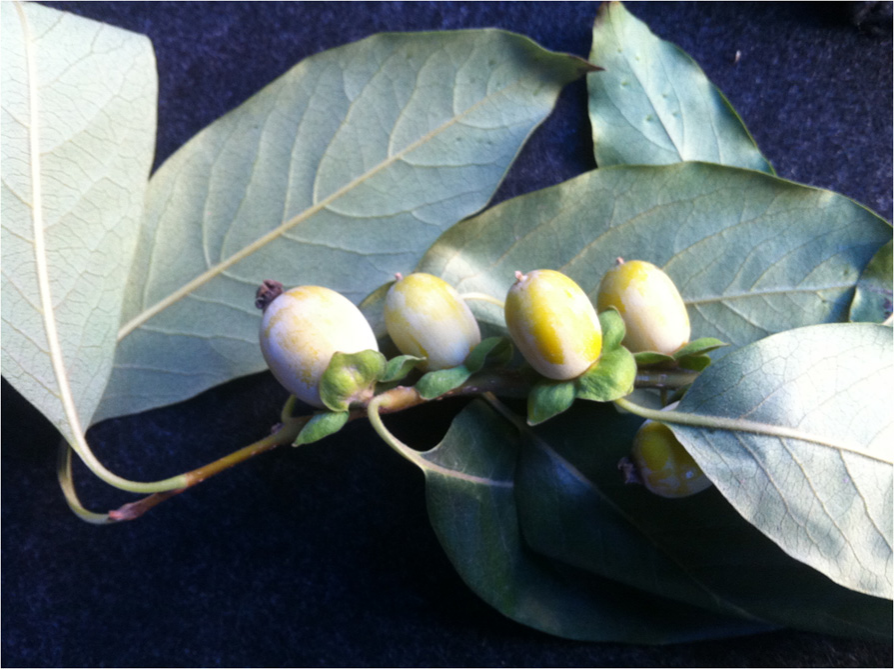
Diospyros Lotus.

**Figure 2:**
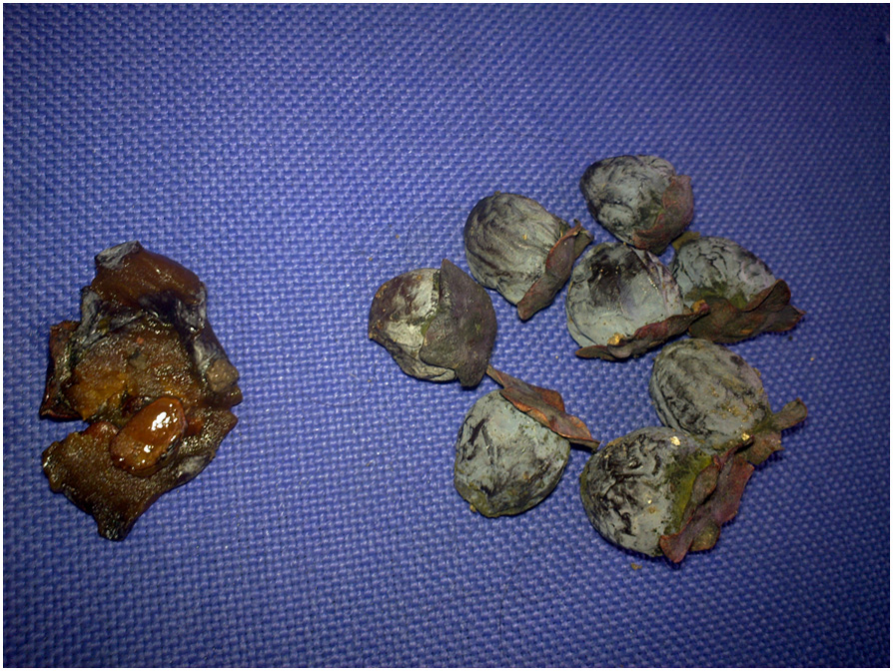
Diospyros Lotus.

Gastrointestinal bezoar is a rarely encountered clinical condition difficult to diagnose and treat. They are classified according to their contents. Phytobezoar is the most common type of gastrointestinal system bezoars that occur due to excessive consumption of herbal nutrients including a high amount of indigestible fibers. Excessive consumption of Diospyros Lotus (Wild Date Palm of Trabzon, Persimmon), which is a traditional nutrient grown particularly in the Black Sea Region of Turkey and includes high amount of indigestible fibers, is thought to be responsible for the high prevalence of gastrointestinal phytobezoars in this region. (Figures 1 and 2**:** Diospyros Lotus)

Clinical picture, ranging from an asymptomatic condition to acute abdomen, depends on the amount of Diospyros Lotus consumed, as well as to the location of phytobezoar.

In addition to radiological imaging methods, upper gastrointestinal endoscopy is used in the diagnosis of phytobezoars.

Prevention is the primary goal in the management of phytobezoars, however when they occur, they have to be removed. Various endoscopic and surgical techniques, including gastric lavage, are used for treatment.

In the present study, the records of 13 patients who had undergone surgical intervention for gastrointestinal phytobezoars, considered to be caused by Diospyros Lotus consumption, were investigated. The aim of the study was to investigate the effects of Diospyros Lotus, which is a widely consumed fruit in our region, together with other predisposing factors on the development of gastrointestinal system phytobezoars, and to discuss the treatment results in comparison to the literature.

## Material and method

The present study was designed as a retrospective study. The medical records of 13 patients, who had been admitted to the General Surgery Clinic of Düzce Atatürk State Hospital between August 2008 and August 2011, and had undergone surgical intervention with a diagnosis of gastric phytobezoar, were reviewed.

Demographic characteristics, predisposing factors, clinical and radiological findings, diagnostic and therapeutic methods were recorded from the patient records, and morbidity and mortality rates were estimated. Current information regarding the disease, such as recurrence, was obtained from the patients themselves, and recorded. Written informed consents were obtained from all patients for publication of this research article and accompanying images.

## Results

Thirteen patients, (84,6% female) with a mean age of 54,4 years, were included in the study.

All the patients had a history of consuming Diospyros Lotus. Ten (76,9%) of these patients had been admitted to the hospital in November and December, harvesting time, when the fruit is highly consumed. The remaining three patients (23%) with a history of consumption dried Diospyros Lotus, had been admitted between March and June. Other predisposing factors included a history of gastric surgery in four (30,7%) patients [Antrectomy and Billroth II Surgery in one (7,6%) and Distal Subtotal Gastrectomy and Billroth II Anastomosis in three (23%) patients], diabetes mellitus, as a concomitant disease, in four (30,7%) patients and dental implants in three (23%) patients. Hypothyroidism, one of the predisposing factors, was identified in none of the patients (Table 1: Predisposing Factors).

**Table 1:** Predisposing Factors

	**n**	**%**
Diospyros Lotus	13	100
History for Gastric Surgery	4	30,7
Diabetes Mellitus	4	30,7
Dental Prosthesis	3	23
Hypothyroidism	__	__

All patients presented to the clinic with extensive abdominal pain, nausea and fecaloid vomiting. Physical examinations of the patients revealed abdominal distension, rigidity, and rebound tenderness, indicating an acute mechanical bowel obstruction.

**Figure 3:**
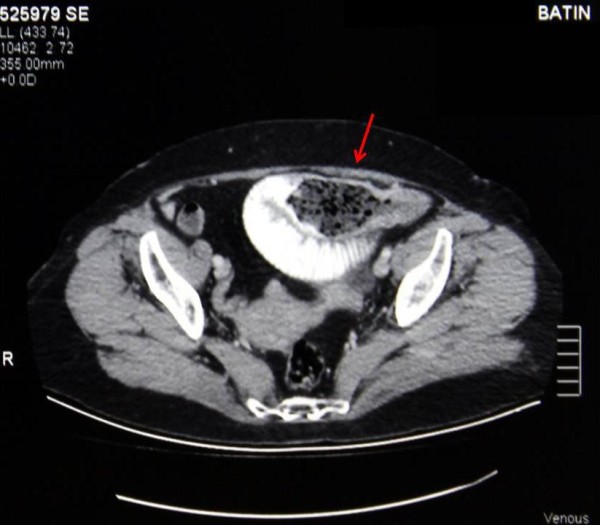
Abdomen Tomography.

**Figure 4:**
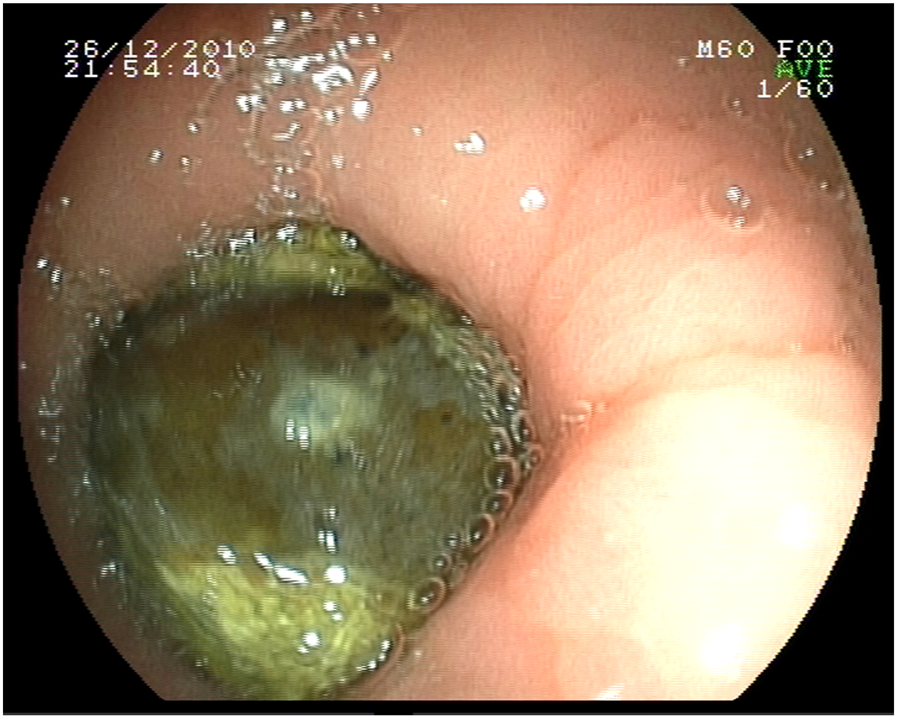
Upper Gastrointestinal Endoscopy.

Plain abdominal radiographs in the standing position showed nonspecific signs such as dilated loops of bowel and air-fluid levels. Diagnosis was based on the abdomen tomography in 11 patients (84,6%), and upper gastrointestinal endoscopy in two (15,3%) patients (Figure 3**:** Abdomen Tomography, Figure 4: Upper Gastrointestinal Endoscopy).

Phytobezoars were found in the stomach alone in three (23%), in the jejunum and stomach in two (15,3%), in the jejunum alone in two (15,3%), and in the ileum alone in six (46,1%) patients (Table 2: Location of Phytobezoars).

**Table 2:** Location of Phytobezoars

	**n**	**%**
Stomach	3	23
Stomach + Jejunum	2	15,3
Jejunum	2	15,3
Ileum	6	46,1

**Figure 5:**
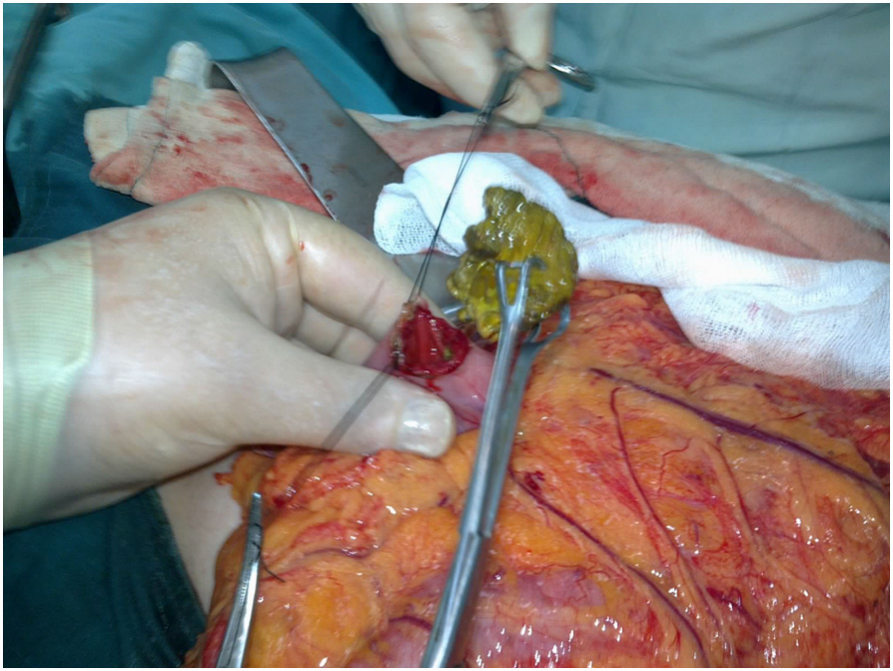
Gastrotomy.

**Figure 6:**
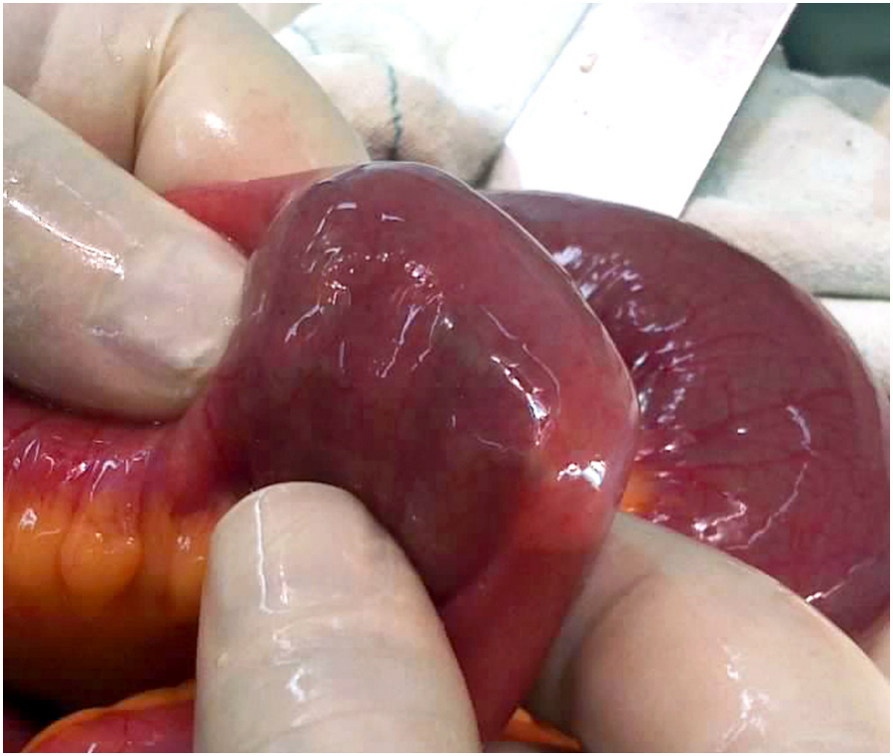
Manual Fragmentation and Milking into Cecum.

All patients underwent surgical intervention including gastrotomy in three (23%), gastrotomy together with manual fragmentation and milking into cecum in two (15,3%), enterotomy in five (38,4%), and manual fragmentation and milking into cecum in three (23%) patients. (Table 3: Surgical Treatment Methods) (Figure 5: Gastrotomy), (Figure 6: Manual Fragmentation and Milking into Cecum).

**Table 3:** Surgical Therapy Methods

	**n**	**%**
Gastrotomy	3	23
Gastrotomy + Manuel Fragmentation and Milking to Cecum	2	15,3
Enterotomy	5	38,4
Manuel Fragmentation and Milking to Cecum	3	23

Pathological examinations were performed. Macroscopically, the material was composed of plant fibers with the seed of Diospyros Lotus at the center. Microscopic examination revealed no cellular elements, but a material composed of plant fibers and food residue.

Only one (7,6%) patient developed wound site infection, which was treated with broad-spectrum antibiotics and daily dressings. None of the patients died.

The mean length of hospital stay was 10,5 days (range, 5–18 days).

The mean postoperative follow-up period was 21,3 months (range, 6–36 months), and no recurrence was observed.

## Discussion

Gastrointestinal bezoars are classified according to their contents. Phytobezoars are the most common type of bezoars, formed by excessive consumption of herbal nutrients. Celery, grape, prune, Diospyros Lotus and pineapple are the main nutrients responsible for phytobezoars. Such nutrients contain high amounts of indigestible fibers, such as cellulose, hemicellulose, lignin and fruit tannins. Trichobezoars, composed of hardened hair and hair-like fibers, are usually encountered in children with mental retardation and in adults with mental illness. Lactobezoar occurs in low birth weight infants fed with concentrated milk and formulas in the first week of life, pharmacobezoar occurs due the use of concentrated drug formulas (cholestyramine and kayexalate); and food bezoars occur due to the use of concentrated food formulas [1-5].

Various predisposing factors are responsible for gastrointestinal phytobezoars; the most important predisposing factors are loss of pyloric function, decreased gastric motility and acid secretion following gastric surgery, adhesions due to abdominal surgery, inadequate chewing, and excessive consumption of herbal nutrients including high amounts of indigestible fibers [3,5-9]. Furthermore, delayed gastric emptying, which results from diabetic neuropathy, hypothyroidism, and connective tissue diseases, forms a basis for the development of gastrointestinal phytobezoars[9-11]. Chisholm et al. retrospectively examined 13 patients with phytobezoars, and found that all the patients had a history of persimmon consumption, whereas 11 (84,6%) had a history of gastric surgery [12]. Krausz et al., in their retrospective study on 113 patients, showed that 106 (93,8%) patients had undergone gastric surgery, whereas 103 (91,1%) had a history of persimmon consumption [10]. In the present study, all 13 patients (100%) had a history of Diospyros Lotus consumption, whereas four (30,7%) had a history of previous gastric surgery. Furthermore, four (30,7%) patients had diabetes mellitus and three (23%) had a history of using dental implants.

The main clinical symptoms are abdominal pain, epigastric distress, nausea and vomiting. In addition, sensation of fullness, dyspepsia, dysphagia, anorexia, weight loss, and gastrointestinal bleeding may be seen [1,13-15]. Decreased bowel sounds, rebound tenderness, rigidity, distension, diarrhea, constipation, nausea and vomiting may be seen in complicated cases [10].

Small bowel obstruction is the most common major complication of phytobezoars. Moreover, gastritis, ulcer, and gastric perforation may be seen. Small bowel phytobezoars usually occur due to the extension of gastric phytobezoars [10,16]. However, small intestinal phytobezoars may also be seen in patients with underlying diseases, such as diverticulitis, stricture, and tumor [17-19]. Small bowel obstructions due to phytobezoars usually occur in the terminal ileum and jejunum, which are the narrowest parts of the small intestine [20]. Chisholm et al. identified phytobezoars in the stomach in two (12,5%), in the jejunum in four (25%), in the ileum in nine (56,2%), and in more than one region of the small intestine in two (12,5%) patients[12]. Krausz et al. detected phytobezoars in the stomach in 13 (11,5%), in the small intestine and stomach in 20 (17,6%), and in the small intestine in 80 (70,7%) patients[10]. In the present study, phytobezoars were located in the stomach alone in three (23%), in the jejunum and stomach in two (15,3%), in the jejunum alone in two (15,3%), and in the ileum alone in six (46,1%) patients.

Upper gastrointestinal endoscopy and radiological imaging methods, such as plain abdominal radiography in the standing position, barium enema radiograph, abdominal ultrasound and abdominal computed tomography, are used for the diagnosis of gastrointestinal phytobezoars. Plain abdominal radiographs may show dilated intestinal loops, air-fluid levels and thickened intestinal wall [17]. Barium radiography is contraindicated in patients with suspected complete obstruction and perforation. Phytobezoars may appear as an echogenic intraluminal mass and a remarkable posterior acoustic shadowing on abdominal ultrasound [21-23]. A dilated small bowel loop with a well-defined, round-shaped, heterogeneous, intraluminal mass distally, is typical on abdominal computed tomography. It typically appears as an intraluminal soft tissue mass that contains air bubbles [9,17,24,25]. Upper gastrointestinal endoscopy can detect all of the gastric phytobezoars, but just 12% of the small bowel phytobezoars[26]. In the present study, diagnosis was made by abdominal tomography in 11 (84,6%), and upper gastrointestinal endoscopy in two patients.

Gastric lavage, and endoscopic or surgical techniques, can be used in the treatment of gastrointestinal phytobezoars.

L-cysteine, metoclopramide and cellulose, papain and cellulose, pineapple juice, normal saline solution, sodium bicarbonate, hydrochloric acid, pancrelipase, pancreatin, 1-2% zinc chloride, and coca cola are used for the disintegration of the bezoar during gastric lavage [3,19,27-29]. Hayashi et al. observed that there was a significant decrease in the size and a significant softening in the structure of the phytobezoar by giving 500–1000 ml coca cola before each meal for three weeks, and they removed the mass using endoscopic forceps [30].

The first successful outcomes concerning endoscopic removal of gastric phytobezoars were published in 1972 by McKechnie[31]. Endoscopic disintegration requires normal pyloric function and absence of duodenal obstruction [27]. If the phytobezoar is not large in size, it can be removed using a basket catheter or by direct aspiration [25].

Surgical therapy may be performed either by open or laparoscopic technique. Main surgical techniques include manual fragmentation and milking to cecum, gastrotomy, enterotomy, and resection and anastomosis in complicated cases. As the prevalence of concurrent gastric and small intestine phytobezoars is 17-21%, care should be given not to leave any residue during surgery [32,33]. Chisholm et al. performed endoscopic removal in one (6,2%), gastrotomy together with manual fragmentation and milking into cecum in one (6,2%), manual fragmentation and milking into cecum in nine (56,2%), enterotomy in four (25%), and small intestine resection and anastomosis in one (6,2%) patient [12]. In a study conducted by Krausz et al., 14 (12,3%) patients underwent gastrotomy, 62 patients (54,8%) underwent manual fragmentation and milking into cecum, 34 patients (30%) underwent enterotomy, and two patients (1,7%) underwent small intestine resection and anastomosis [10]. In the present study, three patients (23%) underwent gastrotomy, two patients (15,3%) underwent gastrotomy together with manual fragmentation and milking into cecum, five patients (38,4%) underwent enterotomy, and three patients (23%) underwent manual fragmentation and milking into cecum.

Krausz et al. reported early morbidity and mortality rates as 11,5% and 1,7%, respectively [10]; the morbidity rate was 7,6% in the present study, whereas no mortality was observed. 

## Conclusion

In conclusion, gastrointestinal phytobezoar is a rare clinical condition, difficult to treat and diagnose. Prevention is the best way to manage the disease. Therefore, excessive consumption of herbal nutrients, containing high amounts of indigestible fibers, such as Diospyros Lotus should be avoided by people with a history of gastric surgery or poor oral and dental health.

### Consent

Written informed consents were obtained from all patients for publication of this research article and accompanying images. A copy of the written consent is available for review by the Editor-in-Chief of this journal.

## Competing interests

I declare that I have no competing interests.

## **Authors’ contributions**

G E, M C, B Y and F E performed the surgeries. G E, M S and T T analyzed and interpreted the data. G E was the main author of the manuscript. All authors read and approved the final manuscript.
